# Dissemination and Refutation of Rumors During the COVID-19 Outbreak in China: Infodemiology Study

**DOI:** 10.2196/22427

**Published:** 2021-02-15

**Authors:** Bin Chen, Xinyi Chen, Jin Pan, Kui Liu, Bo Xie, Wei Wang, Ying Peng, Fei Wang, Na Li, Jianmin Jiang

**Affiliations:** 1 Department of Tuberculosis Control and Prevention Zhejiang Provincial Center for Disease Control and Prevention Hangzhou China; 2 School of Public Health Fudan University Shanghai China; 3 School of Medicine Department of Preventative Medicine Ningbo University Ningbo China; 4 Department of Non-communicable Disease Prevention Zhejiang Provincial Center for Disease Control and Prevention Hangzhou China; 5 School of Urban Design Wuhan University Wuhan China; 6 Department of Public Health Emergency Response Zhejiang Provincial Center for Disease Control and Prevention Hangzhou China; 7 Key Laboratory of Vaccine Prevention and Control of Infectious Disease of Zhejiang Province Hangzhou China

**Keywords:** rumor, Internet, COVID-19, epidemic, misinformation, infodemiology, infodemic, social media, communication, public health

## Abstract

**Background:**

During the outbreak of COVID-19, numerous rumors emerged on the internet in China and caused confusion among the public. However, the characteristics of these rumors in different phases of the epidemic have not been studied in depth, and the official responses to the rumors have not been systematically evaluated.

**Objective:**

The aims of this study were to evaluate the rumor epidemic and official responses during the COVID-19 outbreak in China and to provide a scientific basis for effective information communication in future public health crises.

**Methods:**

Data on internet rumors related to COVID-19 were collected via the Sina Weibo Official Account to Refute Rumors between January 20 and April 8, 2020, extracted, and analyzed. The data were divided into five periods according to the key events and disease epidemic. Different classifications of rumors were described and compared over the five periods. The trends of the epidemic and the focus of the public at different stages were plotted, and correlation analysis between the number of rumors and the number of COVID-19 cases was performed. The geographic distributions of the sources and refuters of the rumors were graphed, and analyses of the most frequently appearing words in the rumors were applied to reveal hotspots of the rumors.

**Results:**

A total of 1943 rumors were retrieved. The median of the response interval between publication and debunking of the rumors was 1 day (IQR 1-2). Rumors in text format accounted for the majority of the 1943 rumors (n=1241, 63.9%); chat tools, particularly WeChat (n=1386, 71.3%), were the most common platform for initial publishing of the rumors (n=1412, 72.7%). In addition to text rumors, Weibo and web pages were more likely to be platforms for rumors released in multimedia formats or in a combination of formats, respectively. Local agencies played a large role in dispelling rumors among social media platforms (1537/1943, 79.1%). There were significant differences in the formats and origins of rumors over the five periods (*P*<.001). Hubei Province accounted for most of the country’s confirmed rumors. Beijing and Wuhan City were the main centers for debunking of disinformation. The words most frequently included in the core messages of the rumors varied by period, indicating shifting in the public’s concern.

**Conclusions:**

Chat tools, particularly WeChat, became the major sources of rumors during the COVID-19 outbreak in China, indicating a requirement to establish rumor monitoring and refuting mechanisms on these platforms. Moreover, targeted policy adjustments and timely release of official information are needed in different phases of the outbreak.

## Introduction

In December 2019, an outbreak of COVID-19, caused by infection with SARS-CoV-2, emerged in Wuhan, Hubei Province, China, and subsequently became a global pandemic [1-3]. As of July 1, 2020, more than 10.3 million cases had been confirmed in most countries and territories worldwide, with more than 508,000 fatalities [4], seriously threatening the lives and health of the public and jeopardizing the stable economic development and social safety of nations.

Internet public opinion crises characterized by frequent rumors often accompany public health emergencies, especially when information from official authorities is delayed or lacking [5]. Since the advent of Web 2.0 technology, internet social media platforms such as WeChat (similar to WhatsApp) and Weibo (similar to Twitter) have gradually replaced traditional media as the main platforms for the public to express their opinion and participate in social affairs in China. Due to the easy accessibility and convenience of social media, information spreads more rapidly and widely through these platforms than through their conventional counterparts [6]; moreover, the resulting large availability of user-provided content fosters massive recruitment of people around common interests, worldviews, and narratives, thus affecting the evolution of public opinion [7] and further enabling rumors to flourish. In 2013, the World Economic Forum described web-based rumors as “digital wildfire” and highlighted the risks they pose to modern society [8]. The rumor that drinking spirit can prevent infection with SARS-CoV-2 is a typical example of a rumor that spread during the outbreak of COVID-19 in China. From the beginning of the outbreak, social media users started to query about methods of preventing and treating COVID-19, and they rushed to the internet to seek information. Due to strong concerns about their own lives and the lack of awareness of the disease, many microbloggers released messages that misrepresented the causal relationship between COVID-19 and drinking spirit, and their posts became very prevalent [9]. This false message was widely discussed on the internet at the time and caused great confusion and panic. As a result, the government provided an official clarification of the rumor, and various localities promptly refuted the rumor and addressed it through the intervention of public security departments, which prevented the rumor from spreading further.

According to previous research, public opinion events are often caused by the interaction of events, the public, social media platforms, and structural factors of the government [10,11]. Public health emergencies, especially outbreaks of new infectious diseases, are often accompanied by uncertainty about the cause of the emergency. However, the resulting information on the morbidity and mortality of the diseases becomes the focus of public concern from the moment it emerges. Individuals’ perceptions of the threat of diseases tends to be reinforced by their exposure to case data and also by public and private information that is disseminated widely on social media [12]. Simultaneously, the unknown causes of public health emergencies stimulate increased information-seeking behavior in people who are aiming to reduce their uncertainties about the emergent situation [13]. However, in the absence of information, people experience a wide range of emotions in the face of unexpected situations, and the anxiety or fear thus generated can exacerbate the occurrence and dissemination of rumors [14]. The role of government intervention in the development trend of rumors remains uncertain. However, the subject, duration, methods, and level of government intervention have certain influences on the virality of rumors [10]. In addition to the professional measures of epidemic prevention and control, keeping the information accurate and transparent and preventing the spread of rumors are critical parts of the crisis response, reflecting the significance of the establishment of government monitoring-feedback-intervention mechanisms in public health emergencies [15].

Compared with the severe acute respiratory syndrome (SARS) outbreak 17 years ago, the COVID-19 outbreak has sparked more rumormongering. Rumors such as “dual yellow oral liquid inhibited novel coronavirus,” “number of confirmed cases of COVID-19 and deaths in a county,” and “some places have been blockaded or the supermarkets have been closed down” sparked panic among the public, causing people to rush to buy supplies and posing a serious challenge to the governance of internet public opinion in the context of the epidemic. As the challenges grew in the face of the public crisis, the phenomenon of the “infodemic,” an overabundance of accurate or inaccurate information occurring during an epidemic, has escalated to a level that requires a coordinated response. Thus, the emerging research area of “infodemiology,” which can be defined as the science of using epidemiologic methods and terminology to study the distribution and determinants of information in an electronic medium, specifically the internet, with the ultimate aim to inform public health and public policy, has been developed [16] and was effectively used to predict the influenza outbreak in 2006 [17]. Infodemiology data are derived from unstructured, textual, openly accessible information produced and consumed by the public on the internet to demonstrate and explore the opinions, focus, behavior, attitudes, and knowledge of the public [16]. The research field of infodemiology has gradually gained wider use, and it caught the attention of the World Health Organization in the wake of the COVID-19 outbreak [18], encouraging the undertaking of more relevant studies and effective practices to understand more about internet information.

Thus, in this study, we analyzed rumors collected from rumor-refuting platforms, using the methods of infodemiology from the supply side, to understand the epidemic of rumors and official responses in different periods according to typical events and the disease epidemic. The results of this study could provide evidence-based recommendations for information communication and rumor prevention during subsequent public health emergencies.

## Methods

### Data Sources

Between January 20, 2020 (the day the national authority first published the official announcement of human-to-human transmission of COVID-19), and April 8, 2020 (the day the lockdown was lifted in Wuhan), daily reports of identified and confirmed rumors and counterrumors of the COVID-19 outbreak were collected through the Sina Weibo Official Account to Refute Rumors (hereinafter referred to as "the Rumors on Weibo account") [19]. Launched in 2010, this account has been dedicated to rumor-busting and has now become one of the largest accounts in China, with more than 2.16 million registered users and subscribers; it contains a massive amount of information collected from most official platforms, such as the Chinese National Platform to Refute Rumors, WeChat Disinformation Platform, internet media, and web pages of government authorities, to refute rumors. Each post in the Rumors on Weibo account contains a rumor message and related rumor-refuting information.

**Figure 1:**
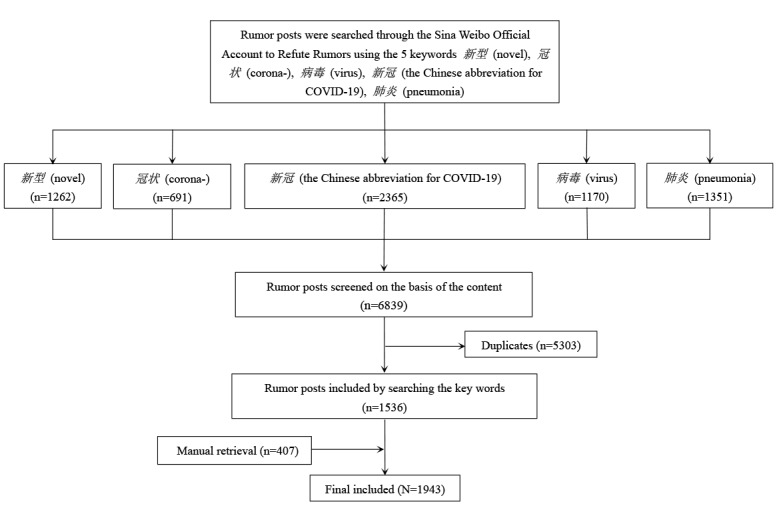
Flowchart of the selection of rumor posts.

During the first week of the study period (January 20-27), a small sample of 311 rumor messages was collected via the Rumors on Weibo account, and word frequency analysis was performed on these messages. After a panel discussion, *新型* (novel), *冠状* (corona-), *病毒* (virus), *新冠* (the Chinese abbreviation for COVID-19), and *肺炎* (pneumonia) were selected as the 5 keywords according to their frequency in the rumor posts. A total of 6839 disinformation messages were retrieved on the Rumors on Weibo account based on these keywords during the whole study period, of which 5303 were duplicated; eventually, 1536 rumor messages related to the epidemic were included. Meanwhile, other rumor posts not containing the above keywords were reviewed manually in the Rumors on Weibo account each week. A total of 407 relevant rumor messages, not containing the above 5 keywords, were also retrieved and added to the database (Figure 1). The initial posting time, title, posting platform, geographic location, format of each rumor, rumor-refuting time, and number of retweets of each rumor post were extracted and entered into the database. All the information obtained on the web was in simplified Chinese language and released publicly by the websites; however, no personal identification information was collected.

### Classification of the Five Time Periods

**Figure 2:**
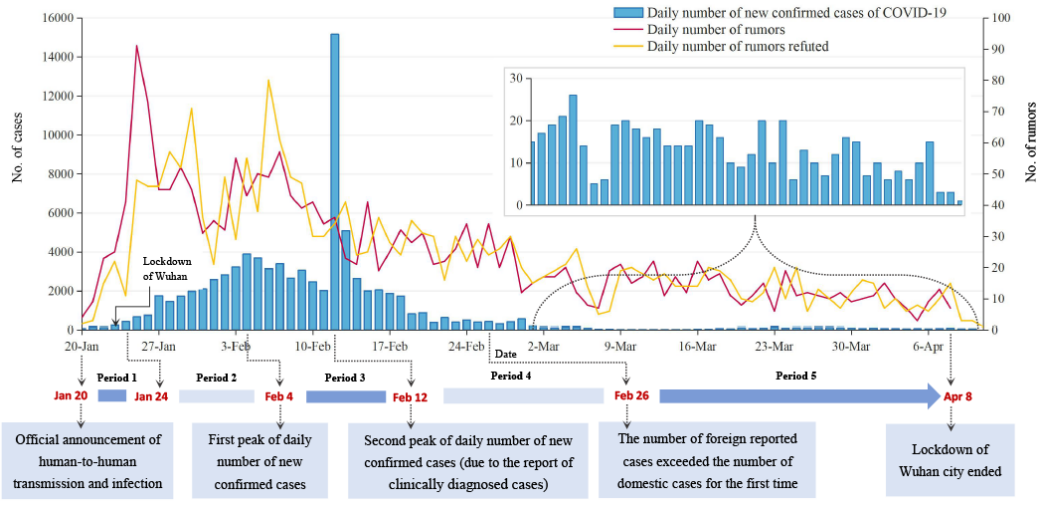
Daily numbers of rumors detected and refuted, the epidemic curve, and key events across five periods of the COVID-19 outbreak in China. The inset shows a magnified view of the number of COVID-19 cases over the last two periods.

To better reflect the dynamics of the spread of rumors, five time periods were classified based on key events of the disease epidemic that could affect the dissemination of rumors on the internet (Figure 2). The time period between January 20, 2020 (the date of the first official announcement of human-to-human transmission and infection of SARS-CoV-2), and January 24, 2020, was taken as the first period in this study [20]. During this period, the National Health Commission issued a bulletin that COVID-19 had been incorporated into the management of the Prevention and Control of Infectious Diseases Act [21], and many local governments had launched first-level responses in the face of the outbreak. On January 23, the Wuhan government blocked all outbound transportation from the city with traffic suspension and home quarantine within the city [22]. The second period was January 24 to February 4; during this time, the number of new confirmed cases in China was gradually rising, and it reached its highest daily increase on February 4 [23]. From February 5 to February 12 (the third period), there was a steady decrease in the daily report of new confirmed cases. However, the secondary peak of the case numbers was reached on February 12 due to reports of clinically diagnosed cases [24]. From then until February 26 (the fourth period), the domestic epidemic gradually declined; meanwhile, foreign cases surpassed the domestic numbers for the first time by February 26. The fifth period was February 27 to April 8, when the domestic epidemic situation continued to decline and the epidemic situation in Wuhan gradually improved. On April 8, Wuhan government announced that the lockdown of the city had ended [25].

### Classification and Definition of Information Sources for Rumors

The rumor information sources were divided into the following categories according to their original publishing platforms: chat tools, Weibo, web pages, and others. Chat tools included WeChat and Tencent QQ. Both of these tools are major social media platforms in China that enable people to chat, communicate, and post events or opinions that others can comment on. If a rumor was first published on Weibo, the Twitter-like platform, which enables users to repost or comment, its information source was classified as Weibo. Web pages represented a number of rumors published in the form of web links. “Others” indicated some platforms that could not be classified in any of the above categories, including several mobile apps, such as TikTok, and platforms on which the source of the rumor could not be traced.

### Classification and Definition of Rumor Refuters

According to the level of representativeness and influence, the rumor refuters were divided into three main categories: national, local, and other. “National” indicated that the official accounts of the rumor refuters were set up by national departments or institutions for publishing official state news or policies. Accounts established by relevant local agencies for publishing local news were classified as local, and other accounts that could not be classified in either of the above categories were classified as others; these users mainly included certified individual accounts, foreign accounts, and certified accounts of nonstate, nonlocal business firms. Meanwhile, based on the essential attributes and affiliations of different rumor refuters, all of them were further classified as government authorities, news media, and organizations, companies, or individuals. All data were classified based on the classification criteria outlined by two researchers. A third reviewer discussed the different classifications with the two reviewers and finalized the categorization. Some sample accounts are listed in Table 1.

**Table 1:** The criteria of the categories for the rumor refuters and the sample accounts.

Category of rumor refuter		Definition	Sample accounts
**Level of representativeness and influence**			
	National	Official accounts set up by national departments or institutions for publishing official state news or policy	CCTV News Chinese National Platform to Refute Rumors China Science Communication
	Local	Accounts established by relevant local agencies for publishing local news	Wuhan Release Nanjing Release Shanghai Customs
	Others	Accounts that could not be classified into the categories above	Alipay Doctor Guan
**Essential attributes and affiliations**			
	Government authorities	Official accounts established by government departments for the publication of government-related information	Wuhan Release Fuyang Health Committee Jiangxi Public Security Department
	News media	Official accounts of news media websites	CCTV News Wuhan Daily News People’s Daily
	Organizations/companies/individuals	Accounts of some nongovernmental organizations and corporate and individual accounts.	Beijing Public Transportation group Ding Talk Alipay

### Analysis of Most Frequently Appearing Words in Rumors

Rumor word frequency analysis was carried out using Python version 2.7. The jieba package was employed as a text analysis tool for processing the core message of each rumor. The core message of every rumor in our study was summarized by the Rumors on Weibo account, which captured the main point of the content of the entire rumor well. Additionally, the rumors presented in the forms of images, videos, and audio were also translated by extracting the core message of that rumor through the official rumor-refuting account. Accordingly, all the rumor information in this study consisted of brief textual sentences. First, the spaces and newlines were removed from the text; then, all the punctuation marks in the text were replaced with spaces. The processed text was partitioned using the jieba package, and stop words such as “have,” “is,” “will,” “can,” and “have been” were removed from the text according to a predefined list of deactivated words. After the data set was created, a corpus of rumors was formed.

Based on the collection and classification of the rumor corpus obtained by preprocessing, the lists of words were filtered and merged according to an expert’s opinion, and the data set of rumor features was finally obtained. The weight of every term was calculated using the term frequency–inverse document frequency (TF-IDF) method using the equation below:

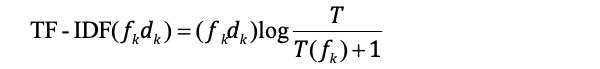



The formula is divided into two parts; (*f_k_d_k_*) to the left of the equal sign represents the word frequency, which is the number of times a word appears in the text. The higher the number of occurrences of a word, the greater the role it plays in the text. In contrast, (*f_k_d_k_*) to the right of the equals sign is the logarithm value of the inverse document frequency. *T* represents the number of texts in the corpus, and *T*(*f_k_*) indicates the number of texts containing specific terms in the corpus.

The word cloud of rumors about COVID-19 during the whole study period was visualized using the wordcloud package; moreover, the summary of the top 20 high-frequency keywords of the rumors based on the term frequency–inverse document frequency (TF-IDF) values over each period was used to analyze the changes in the same keyword at different stages, which may further reflect the shifts in the public’s focus.

### Statistical Analysis

Excel (Microsoft Corporation) was used to record and sort information about the rumors. The epidemic curve, daily number of rumors released and clarified, new confirmed cases of COVID-19, and key events across the five periods were plotted to comprehensively analyze the relationship between the epidemic and the public’s focus at different stages. Descriptive analysis of the basic information of the rumors was conducted using SPSS for Windows, version 24.0.0 (IBM Corporation). The geographic distributions of rumor sources and refuters were graphed using the pyecharts package in Python version 2.7. In addition, the cumulative number of cases and rumors in each province was calculated. The Pearson chi-square test and Fisher exact test were performed to compare different characteristics of the rumors by each category across the five periods. The Spearman rank correlation coefficient was used to explore the relationship between the number of rumors and the epidemic trends if the variables did not satisfy the normal distribution. *P* values were 2-tailed, with statistical significance set at .05.

## Results

A total of 1943 rumors were collected from the Rumors on Weibo account in this study between January 20 and April 8, 2020.

### Characteristics of Rumors According to the Five Periods

The numbers of rumors published across the periods were 102, 547, 349, 377, and 568, respectively (Table 2). Rumors in the form of texts were predominant (1241/1943, 63.9%), accounting for more than half of the rumors in each time period, followed by rumors in a combination of two or more formats (330/1943, 17.0%). Among the rumor-spreading platforms, chat tools were the most common (1412/1943, 72.7%), with 1386/1943 rumors circulating in WeChat, accounting for the vast majority (98.2%). The proportions of the 1943 rumors circulating from Weibo (n=180, 9.3%), web pages (n=162, 8.3%), and other platforms (n=189, 9.7%) were similar. Additionally, 19.4% (376/1943) and 79.1% (1537/1943) of the rumors were refuted by national and local agencies, respectively. Most rumors were clarified by relevant government authorities (1250/1943, 64.3%), followed by the news media (628/1943, 32.3%).

The epidemic curve and daily number of posted and refuted rumors graphed according to the key events are illustrated in Figure 2. Spearman rank coefficient analysis showed that the daily number of posted rumors was positively associated with the daily number of new confirmed cases (Spearman rank correlation coefficient 0.73, *P<*.001). The median of the response interval between the time when the rumors were initially published and debunked was 1 day, with an IQR of 1-2. Most rumors detected were mainly concentrated between January 24 and February 7, while the majority of the refuting posts were concentrated between January 25 and February 9, with the highest daily reports of posting and refuting rumors occurring on January 25 (n=91) and February 6 (n=80), respectively.

**Table 2:** Characteristics of rumors across five periods during the outbreak of COVID-19 in China (N=1943).^a^ *P*<.001 for all categories.

Characteristic		Time periods (2020)					Total (N=1943)	*χ*^2^ (*df*)
		Jan 20-24 (n=102)	Jan 25-Feb 4 (n=547)	Feb 5-12 (n=349)	Feb 13- 26 (n=377)	Feb 27-Apr 8 (n=568)		
**Format of rumor, n (%)**								41.6 (12)
	Text	69 (67.6)	354 (64.7)	228 (65.3)	228 (76.4)	362 (63.7)	1241 (63.9)	
	Picture	6 (5.9)	26 (4.8)	18 (5.2)	17 (4.5)	47 (8.3)	114 (5.9)	
	Multimedia^b^	6 (5.9)	85 (15.5)	38 (10.9)	41 (10.9)	88 (15.5)	258 (13.3)	
	Combination^c^	21 (20.6)	82 (15.0)	65 (18.6)	91 (24.1)	71 (12.5)	330 (17.0)	
**Initial platform of rumor posting, n (%)**								127.6 (12)
	Chat tools^d^	75 (73.5)	453 (82.8)	270 (77.4)	279 (74.0)	335 (59.0)	1412 (72.7)	
	Weibo	15 (14.7)	39 (7.1)	41 (11.7)	30 (8.0)	55 (9.7)	180 (9.3)	
	Web pages	10 (9.8)	29 (5.3)	11 (3.2)	29 (7.7)	83 (14.6)	162 (8.3)	
	Others^e^	2 (2.0)	26 (4.8)	27 (7.7)	39 (10.3)	95 (16.7)	189 (9.7)	
**Level of rumor refuter, n (%)**								61.0 (8)
	National	29 (28.4)	65 (11.9)	63 (18.1)	77 (20.4)	142 (25.0)	376 (19.4)	
	Local	66 (64.7)	478 (87.4)	280 (80.2)	295 (78.2)	418 (73.6)	1537 (79.1)	
	Others	7 (6.9)	4 (0.7)	6 (1.7)	5 (1.3)	8 (1.4)	30 (1.5)	
**Type of rumor refuter, n (%)**								219.5 (8)
	Government authority	67 (65.7)	466 (85.2)	240 (68.8)	217 (57.6)	260 (45.8)	1250 (64.3)	
	News media	25 (24.5)	74 (13.5)	101 (28.9)	152 (40.3)	276 (48.6)	628 (32.3)	
	Organization, company, or individual	10 (9.8)	7 (1.3)	8 (2.3)	8 (2.1)	32 (5.6)	65 (3.3)	

^a^The five periods were classified based on key events and the disease epidemic that could affect the dissemination of rumors on the internet from January 20 to April 8, 2020.

^b^Multimedia: video, audio, and news reports.

^c^Combination: two or three formats were combined to disseminate rumors.

^d^Chat tools: WeChat and Tencent QQ.

^e^Others: other platforms that could not be classified in any of the above categories, including several mobile apps such as TikTok, and platforms that could not be traced back.

### Characteristics of Rumors on Different Posting Platforms

A comparison of the 1943 rumors categorized by rumor-posting platform is shown in Table 3. Text (1241/1943, 63.9%) was the most common rumor format across different posting platforms, while the image format (114/1943, 5.9%) had the lowest percentage of all rumors. In addition to the texts, there were more rumors disseminated in a combination of formats on Weibo (60/180, 33.3%), while web pages (54/162, 33.3%) and others (42/189, 22.2%) tended to be the initial publishing platforms for rumors in multimedia format. Pearson chi-square tests indicated that there were statistically significant differences in the formats of rumors classified by platform (*χ^2^_9_*=142.6, *P*<.001). Local agencies played a large role in dispelling rumors on the rumor-spreading platforms. The rumor refuters of government authorities and news media worked in tandem and complemented each other, and together they dispelled approximately 90% of the rumors on every platform. Fisher exact tests and Pearson chi-square tests suggested that the types of rumor refuters were significantly different across the platforms (*P*<.001).

**Table 3:** Comparison of different rumor posting platforms between January 20 and April 8, 2020 (N=1943). *P*<.001 for all categories.

Classification		Initial platform of rumor posting, n (%)					
		Chat tools^a^ (n=1412)	Weibo (n=180)	Web pages (n=162)	Others^b^ (n=189)	Total (N=1943)	*χ*^2^ (*df)*
**Format of rumor, n (%)**							142.6 (9)
	Texts	941 (66.6)	85 (47.2)	81 (50.0)	134 (70.9)	1241 (63.9)	
	Pictures	91 (6.4)	11 (6.1)	8 (4.9)	4 (2.1)	114 (5.9)	
	Multimedia^c^	138 (9.8)	24 (13.3)	54 (33.3)	42 (22.2)	258 (13.3)	
	Combination^d^	242 (17^.^1)	60 (33.3)	19 (11.7)	9 (4.8)	330 (17.0)	
**Level of rumor refuter, n (%)**							N/A^e^
	National	145 (10.3)	63 (35.0)	81 (50.0)	87 (46.0)	376 (19.4)	
	Local	1256 (89^.^0)	109 (60.6)	73 (45.1)	99 (52.4)	1537 (79.1)	
	Others	11 (0.8)	8 (4.4)	8 (4.9)	3 (1.6)	30 (1.5)	
**Type of rumor refuter, n (%)**							180.2 (6)
	Government authorities	1030 (72.9)	87 (48.3)	56 (34.6)	77 (40.7)	1250 (64.3)	
	News media	354 (25.1)	80 (44.4)	95 (58.6)	99 (52.4)	628 (32.3)	
	Organization/company/individual	28 (2.0)	13 (7.2)	11 (6.8)	13 (6.9)	65 (3.3)	

^a^Chat tools: WeChat and Tencent QQ.

^b^Others: other platforms that could not be classified in any of the above categories, including several mobile apps such as TikTok, and platforms on which the source of the rumors could not be traced.

^c^Multimedia: video, audio, and news reports.

^d^Combination: two or three formats were combined to disseminate rumors.

^e^N/A: not applicable (*P* value was calculated using Fisher exact test).

### Geographic Distribution of Rumor Sources and Refuters

**Figure 3:**
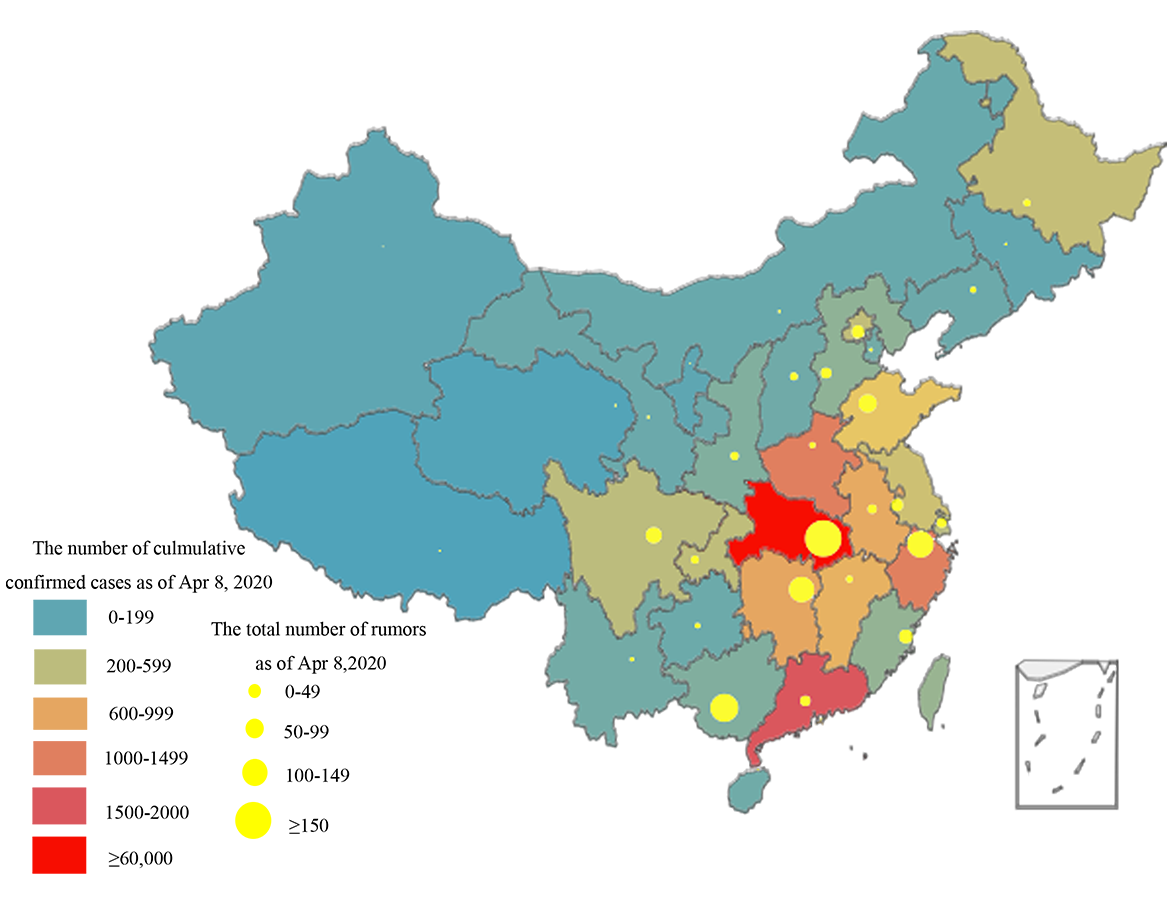
Cumulative numbers of confirmed cases of COVID-19 and rumors in each region of China as of April 8, 2020.

The cumulative numbers of confirmed cases of COVID-19 and rumors generated by province across China as of April 8, 2020, are visualized in Figure 3. The internet rumors spread more actively in the southeast regions. In particular, Hubei Province and its surrounding areas showed the highest numbers of both confirmed cases and rumors in the country. The cumulative number of confirmed cases in Guangdong Province as of April 8 was ranked second in the country, whereas rumor generation in that province was relatively low. Conversely, although Guangxi Province had fewer cases, its number of rumors was among the highest nationwide.

**Figure 4:**
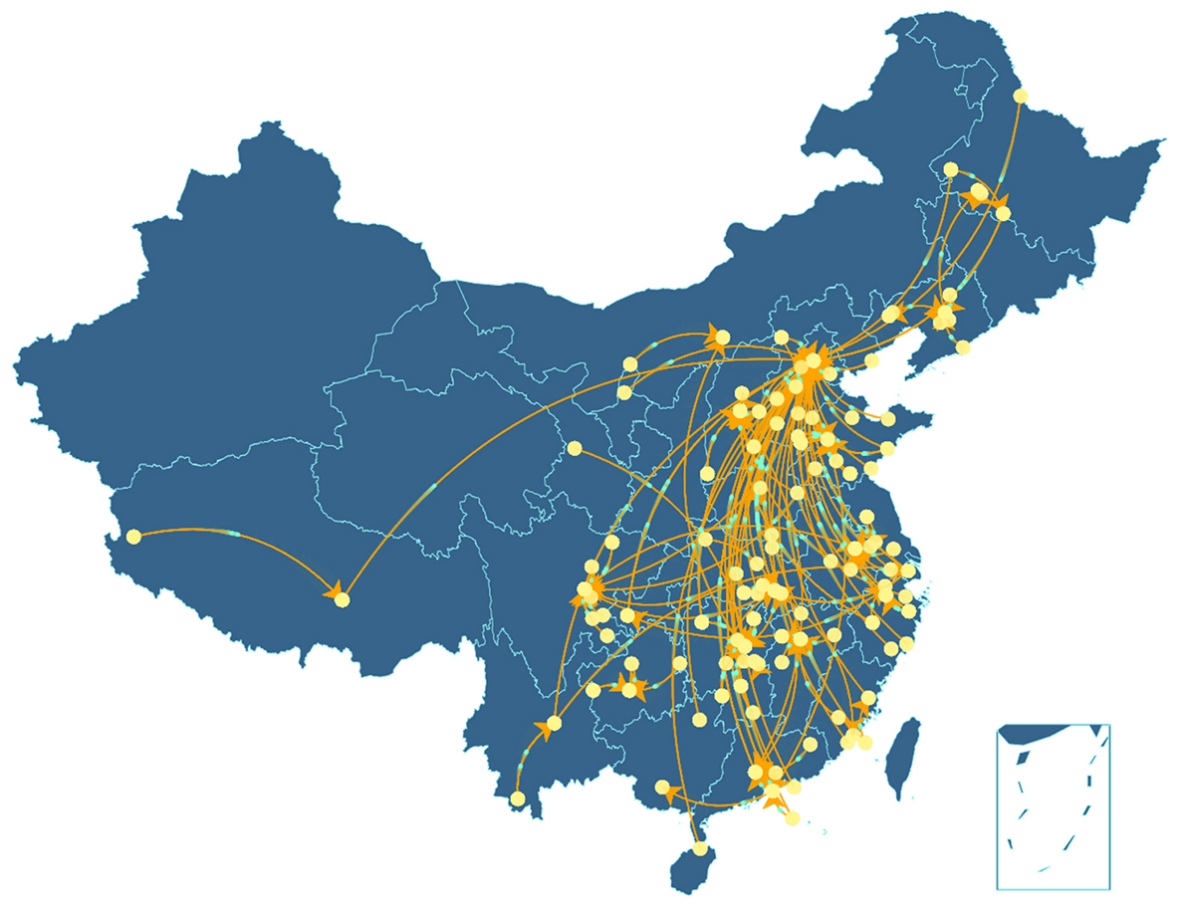
The geographic distributions of rumor sources and refuters during the outbreak of COVID-19 between January 20 and April 8, 2020, in China.

The geographic distributions of the rumor sources and their corresponding refuters are graphed in Figure 4, where the arrow symbols indicate rumor-refuting locations. Beijing and Wuhan were the two main hubs of disinformation refuting during this stage of the COVID-19 epidemic. In Guangdong Province, where the epidemic was relatively severe, more rumors were refuted than spread. Except for the rumors that were clarified locally, rumors circulating within the provinces were often officially refuted by the provincial governments.

### General Focus and Frequent Words of Rumors During the Five Phases

**Figure 5:**
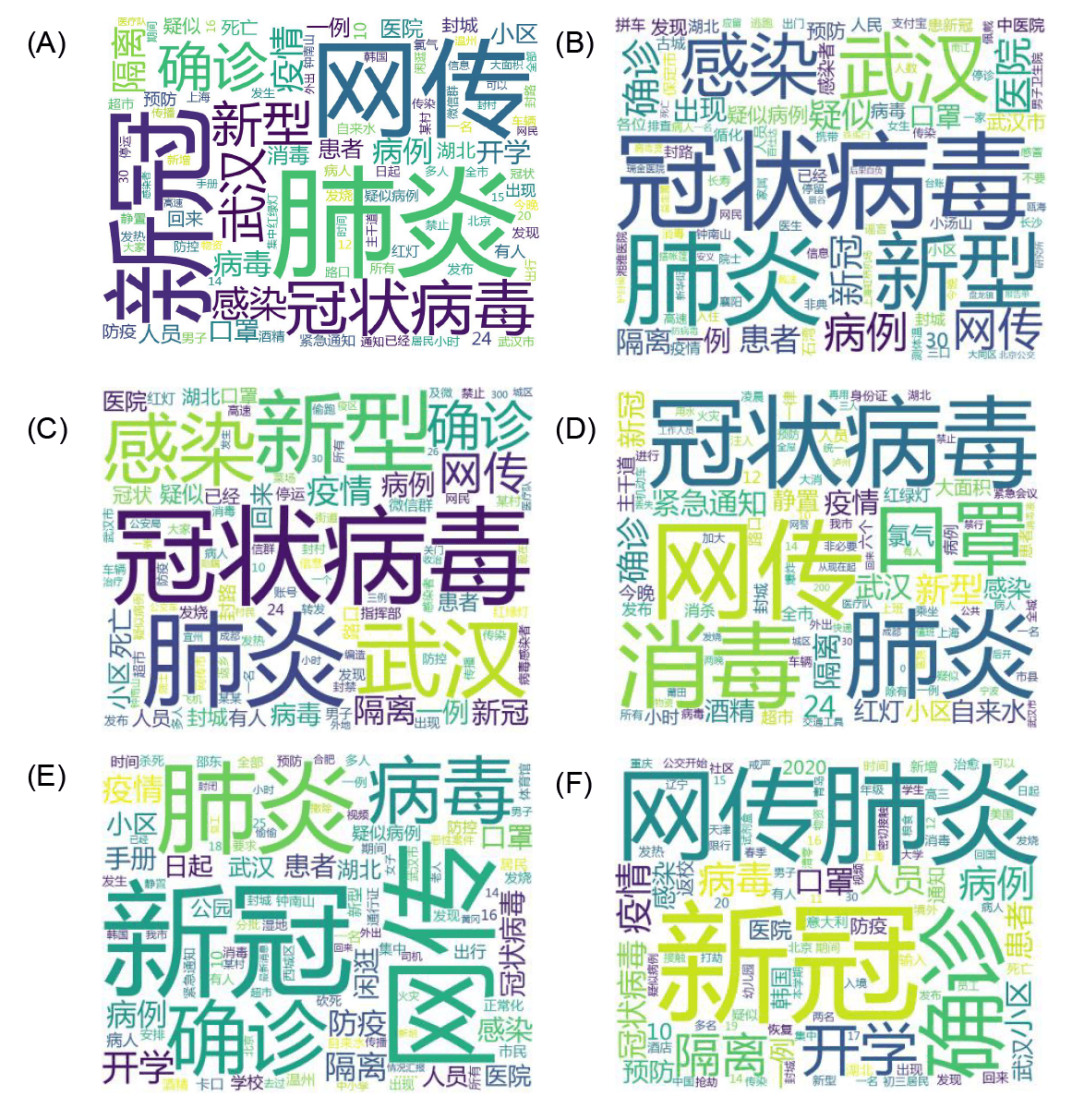
The top 100 most frequent words in the core messages of rumors during the outbreak of COVID-19 in China: (A) during the whole study period, (B) from January 20-24, 2020, (C) from January 25 to February 4, 2020, (D) from February 5-12, 2020, (E) February 13-26, 2020, and (F) from February 27 to April 8, 2020.

The top 100 most frequent words of the core messages of the rumors throughout the study period and different phases of the outbreak are depicted in Figure 5 (also see Multimedia Appendix 1 for a description of the evolution trends of the top 10 most frequent words, excluding the search keywords, at different periods during the outbreak). The average length of the textual rumors was nearly 19 characters. Overall, *冠状病毒* (coronavirus) and *肺炎* (pneumonia) were the two most common words included in the rumor core message. A majority of the words, such as *病毒* (virus), *新型* (novel), *新冠* (the Chinese abbreviation for COVID-19), and *病例* (case), were associated with COVID-19. In addition, *武汉* (Wuhan), *医院* (hospital), *小区* (residential areas), as terms related to specific locations were also frequently found in the rumors. Meanwhile, some words describing epidemic prevention and control, such as *口罩* (mask), *消毒* (disinfection), and *隔离* (quarantine), were often seen.

More details of the top 20 words listed according to their TF-IDF values in the major rumor messages over different periods are presented in Table 4. The top 5 words in the first and second periods were the same. However, the TF-IDF value of each of these words was different; the values in the first stage were higher than those in the second stage. By the third period, *网传* (sourced from internet) had become the top word, while the words *消毒* (disinfection) and *口罩* (mask) had replaced *武汉* (Wuhan) and *感染* (infection) as the fourth and fifth most common words, respectively. In contrast to similar rumors about the epidemic at the other stages, disinformation related to *开学* (schools reopen) began to grow during the fourth and fifth periods. Moreover, as the outbreak of COVID-19 increased outside China, rumors of foreign epidemic situations increased as well.

**Table 4:** The top 20 keywords in rumors across five periods during the outbreak of COVID-19 in China.^a^

Rank^b^	Time periods (2020)									
	Jan 20-24		Jan 25-Feb 4		Feb 5-12		Feb 13-26		Feb 27-Apr 8	
	Keyword	TF-IDF^c^ value	Keyword	TF-IDF value	Keyword	TF-IDF value	Keyword	TF-IDF value	Keyword	TF-IDF value
1	冠状病毒 (coronavirus)	0.480	冠状病毒 (coronavirus)	0.256	网传 (source from internet)	0.157	新冠 (novel corona)	0.251	新冠 (novel corona)	0.283
2	肺炎 (pneumonia)	0.434	肺炎 (pneumonia)	0.237	冠状病毒 (coronavirus)	0.132	网传 (source from internet)	0.219	肺炎 (pneumonia)	0.187
3	新型 (novel)	0.294	新型 (novel)	0.182	肺炎 (pneumonia)	0.123	肺炎 (pneumonia)	0.149	网传 (source from internet)	0.163
4	武汉 (Wuhan)	0.284	武汉 (Wuhan)	0.167	消毒 (disinfection)	0.119	确诊 (confirmed diagnosis)	0.112	确诊 (confirmed diagnosis)	0.122
5	感染 (infection)	0.235	感染 (infection)	0.163	口罩 (mask)	0.085	病毒 (virus)	0.100	开学 (schools reopen)	0.117
6	网传 (source from Internet)	0.151	确诊 (confirmed diagnosis)	0.126	新型 (novel)	0.085	开学 (schools reopen)	0.099	隔离 (quarantine)	0.074
7	病例 (case)	0.127	网传 (source from internet)	0.116	确诊 (confirmed diagnosis)	0.083	隔离 (quarantine)	0.073	病例 (case)	0.070
8	确诊 (confirmed diagnosis)	0.112	疫情 (epidemic situation)	0.102	紧急通知 (urgent notice)	0.079	疫情 (epidemic situation)	0.067	病毒 (virus)	0.069
9	医院 (hospital)	0.111	隔离 (quarantine)	0.095	新冠 (novel corona)	0.076	病例 (case)	0.067	疫情 (epidemic situation)	0.065
10	新冠 (novel corona)	0.090	新冠 (novel corona)	0.077	武汉 (Wuhan)	0.075	防疫 (anti-epidemic)	0.063	人员 (personnel)	0.052
11	疑似 (suspected)	0.081	病例 (case)	0.068	隔离 (quarantine)	0.075	感染 (infection)	0.056	冠状病毒 (coronavirus)	0.046
12	患者 (patients)	0.072	一例 (one)	0.065	疫情 (epidemic situation)	0.067	冠状病毒 (coronavirus)	0.055	口罩 (mask)	0.045
13	隔离 (quarantine)	0.062	病毒 (virus)	0.062	自来水 (piped water)	0.067	口罩 (mask)	0.054	小区 (residential areas)	0.044
14	出现 (appear)	0.058	死亡 (death)	0.059	红灯 (red light)	0.063	闲逛 (hang out)	0.053	患者 (patients)	0.043
15	一例 (one)	0.058	小区 (residential areas)	0.055	酒精 (ethyl alcohol)	0.062	患者 (patients)	0.052	预防 (prevention)	0.042
16	口罩 (mask)	0.057	回来 (back)	0.052	感染 (infection)	0.057	手册 (handbook)	0.051	一例 (one)	0.041
17	疑似病例 (suspected cases)	0.047	医院 (hospital)	0.049	静置 (placed still)	0.055	小区 (residential areas)	0.051	感染 (infection)	0.038
18	预防 (prevention)	0.043	疑似 (suspected)	0.046	氯气 (chlorine)	0.054	日起 (as from today)	0.045	医院 (hospital)	0.030
19	发现 (find)	0.039	患者 (patients)	0.041	小区 (residential areas)	0.054	医院 (hospital)	0.042	武汉 (Wuhan)	0.029
20	病毒 (virus)	0.037	封城 (lockdown)	0.040	大面积 (large tracts of land)	0.053	武汉 (Wuhan)	0.041	韩国 (Korea)	0.028

^a^The five periods were classified based on key events and the disease epidemic that could affect the dissemination of rumors on the internet from January 20 to April 8, 2020.

^b^Keywords are ranked according to the TF-IDF values of the words from high to low.

^c^TD-IDF: term frequency–inverse document frequency.

## Discussion

### Principal Findings

Based on the official Sina Weibo rumor-refuting platform, nearly 2000 rumors that spread over the internet during the COVID-19 epidemic in China between January 20 and April 8, 2020, were investigated. This is the first domestic research to analyze the distribution, characteristics, spreading trend, and most frequent words of rumors related to the epidemic situation, which will be propitious to provide a scientific reference for the prevention and control of network rumors during unexpected events in the future.

During the study period, the median of the response interval was 1 day, indicating the timeliness of the rumor-refuting measures conducted by the Chinese government during the COVID-19 outbreak. In general, the number of rumors and refuted rumors in the first and second periods of the epidemic showed rapid growth, while both showed fluctuating declining trends in the latter three periods. Notably, after the announcement of the Wuhan lockdown, the rumor posts reached their first peak within three days, suggesting that the rapid rise in the number of rumors over the early period has a strong link with the emergence of landmark events. At this stage, due to the sudden outbreak of COVID-19, the etiology and trend of the disease were totally unclear, and the monitoring mechanism for identifying and refuting disinformation had not yet been perfected; thus, the old rumors were not quickly clarified, while new rumors appeared in rapid succession [26]. The new and old rumors intertwined to reach the peak of rumor growth, obfuscating the truth and increasing the difficulty of epidemic prevention and control [27]. By the middle of the epidemic period, most people had developed a preliminary understanding of the disease after obtaining more official information. At this stage, the rumors were less related to symbolic events and were mainly affected by the trend of the epidemic situation, fluctuating with the increase or decrease in the number of cases. Finally, by the time the disease was under control, most people had grasped a more rational understanding of the situation, with the anxiety and tension over the uncertainties greatly alleviated, leading to a decrease of the number of rumors and their corresponding clarifications. Therefore, the early stages of public emergencies, especially new infectious diseases, are the critical period for web-based surveillance of public response, risk communication, and timely release of information from credible sources, as reflected in a study on avian influenza A (H7N9) [28]. Moreover, risk communication will promote community engagement, decrease rumors to maintain social stability, and reduce threats to public health [29]. Intensive information communication with reference to hot topics of rumors may buy time to control outbreaks and reduce the risk of transmission to humans [30,31]. Additionally, transparent sharing of information in time, particularly of adverse information, and projecting uncertainty explicitly are integral parts of the management of large-scale epidemics and other emergencies [32]. Moreover, during the period of steady decline, continuous internet surveillance of rumors is still required.

Different characteristics of the rumors were analyzed in this study; it was found that internet rumors in the early stage of the epidemic were mainly disseminated in text format, most commonly in the WeChat chat tool. More processed and visualized rumors (eg, in the formats of pictures and multimedia) emerged over the later stages, while the number of rumors increased on other platforms, such as Weibo and web pages. The difference in the main formats of rumor dissemination among different platforms may be related to the openness and information screening mechanisms of each platform. In recent years, WeChat has rapidly become the main social platform in China due to its convenience and accessibility. Compared to other social media, WeChat is a social tool based on realistic relationships and closed-loop communication in a relatively private space, implying the reliability and authenticity of information and invisibly increasing the influence of rumors [33,34]. Therefore, when false news circulates, WeChat lacks self-correction ability due to the trust among acquaintances, and it is also more difficult to completely convince WeChat users that a rumor is false even with rumor-dispelling messages on the internet. However, Weibo and web pages are more open and diverse; uncertain information can be questioned, corroborated, corrected, and supplemented through user-produced content, constantly discarding false information and approaching the truth in positive interactions [35,36]. Moreover, Weibo has established an increasingly comprehensive account for refuting rumors [19]. Eventually, through the questioning of netizens, inaccurate information is replaced by the truth. In recent years, WeChat and other platforms have also taken measures to combat rumors [37]. However, the dissemination of refuting information on WeChat is restricted, mainly because it cannot reach the level of interpersonal communication, leading to small-scale transmission; thus, it can only continue to be shown “to people who do not believe rumors” [38]. Thus, a more comprehensive mechanism to encourage the dissemination of rumor-dispelling information should be developed by relevant departments in the future, accelerating the spread of credible information on WeChat and extending the influence of these departments at the same time.

Analysis of the refuters of rumors showed that during the initial outbreak of COVID-19, rumors were likely to be more nationally focused because they were relatively few in number, and the national-level rumor refuting agencies played a stronger role. When the rumors gradually started spreading locally, local authorities increased their rumor-refuting efforts; combined with the increasing enhancement of self-purification of social media, this eased the pressure on government agencies [28]. Similarly, due to the small scope and influence of rumor spread in chat tools, most were clarified by local government authorities. In contrast, rumors circulating in Weibo elicited more involvement and intervention from higher-level authorities, indicating a larger impact. These findings highlight the significance of coordinating the roles of central and local agencies in the establishment of mechanisms for refuting rumors, improving the feedback mechanisms, and maximizing the self-purification ability of social media.

According to the distribution map of the rumors, Hubei Province was the most active area for rumor breeding, which may be related to its highly severe epidemic situation. In this study, locations with more cases were likely to generate more rumors, mainly including cities around Hubei, such as Zhejiang and Hunan. However, rumors also circulated in larger quantities in provinces with lower case numbers, such as Guangxi. Accordingly, the timely identification of rumors in regions less affected by public health emergencies is of equal importance to constant internet surveillance in areas that are more severely affected. The cross-regional rumor-refuting plot shows that Beijing was the critical center for rumor clarification. Shanghai and the provincial capitals of Guangzhou, Hunan, Sichuan, and Zhejiang also played vital roles in dispelling rumors during the epidemic, reflecting to some extent that large cities are political and media centers [39].

The high-frequency words in different periods indicated that in the pre-epidemic period, the rumors were mainly related to the disease itself, with numerous descriptions of COVID-19 contained in the core message of the rumors. In the middle of the epidemic, the rumors gradually began to be associated with prevention and control measures due to the official announcement of epidemic initiatives published by departmental agencies. When the domestic epidemic had been effectively controlled, false information about measures such as school reopening and traffic resumption spread across each region, suggesting that people gradually paid more attention to information related to policy adjustment during this period compared to the previous stages [26]. The word *网传* (source from internet) was frequently included in the titles of rumors. The word *确诊* (confirmed diagnosis) continued to appear frequently across different periods, even when the number of daily new confirmed cases had decreased since the third period. Further, when the words *确诊* (confirmed diagnosis) and *病例* (case) were combined in a rumor, the title usually took the form of “multiple cases have been confirmed in a certain place”; thus, the rumors were in a “storytelling” form that was immersive and highly convincing to readers. Such rumors have also been reported in other studies on infectious diseases [28], providing a reference for accurate identification of rumors in the future.

### Limitations

There were several limitations to this study. First, this study was a retrospective analysis based on information extracted from an official rumor-refuting account, and it was difficult to avoid omitting some detailed information. In some cases, we could not trace or confirm the platforms or geographic locations where the rumors were initially published. Second, because the information of rumors in this study was refined, the emoticons and function words in the original text were not addressed; this could be considered in future research. Third, not all the rumors could be refuted on this account; instead, the most socially influential rumors were included, which could lead to information loss from the less influential rumors.

### Conclusions

Our findings indicate the significance of timely management of and responses to internet rumors during major public crises. In this wave of the COVID-19 outbreak, authorities have taken effective measures to quickly dispel rumors; however, more effort could be made to better address the rumors. WeChat and other chat tools were found to be the most common origins of rumors, suggesting that the early detection and debunking mechanisms of rumors should be strengthened in closed-loop communication environments. In the early stages of the event, authorities should focus on rumors in the form of texts but should also pay more attention to other forms such as multimedia as the event progresses. The words most frequently included in the core messages of the rumors varied over different periods, which may be related to the disease itself, prevention and control measures, and social recovery; this highlights that targeted policy adjustments and timely release of official information in different phases of the outbreak should be required to prevent dissemination of internet rumors. Spread of rumors across borders needs to be controlled regardless of the intensity of the epidemic in the area. Local and national authorities should strengthen joint communication and collaboration in refuting rumors and establish a cooperative refuting mechanism based on the division of functions.

## Appendix

Multimedia Appendix 1 The evolution trends of the top 10 buzzwords (excluding the search keywords) at different periods during the outbreak.

## Abbreviations

H7N9: avian influenza A
SARS: severe acute respiratory syndrome
TF-IDF: term frequency–inverse document frequency
