# Supplementary figures and images for: Dissemination and Refutation of Rumors During the COVID-19 Outbreak in China: Infodemiology Study
Source: J Med Internet Res. 2021 Feb 15;23(2):e22427. doi: 10.2196/22427 (PMC7886374; doi:10.2196/22427)

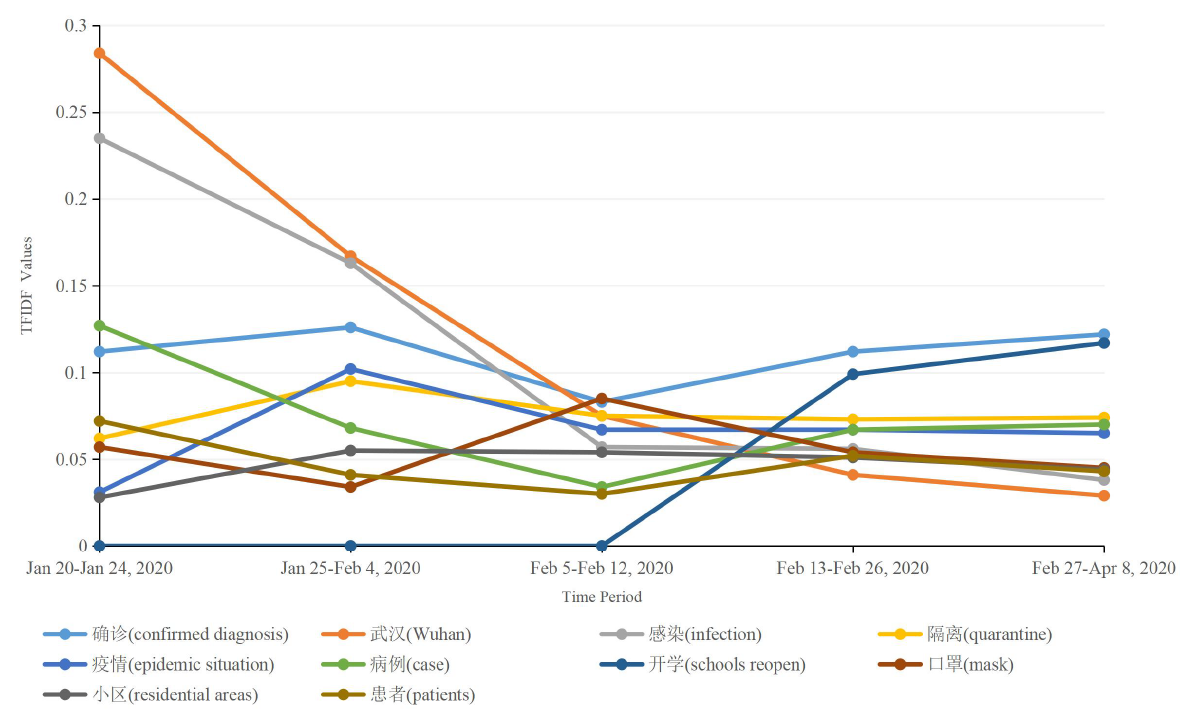

Supplement: Multimedia Appendix 1 [file jmir_v23i2e22427_app1.png]
